# Health Risk Assessment for Potential Toxic Elements in the Soil and Rice of Typical Paddy Fields in Henan Province

**DOI:** 10.3390/toxics12110771

**Published:** 2024-10-23

**Authors:** Yuling Jiang, Hao Guo, Keying Chen, Xiaowei Fei, Mengzhen Li, Jianhua Ma, Weichun He

**Affiliations:** 1 School of Geographic Sciences, Xinyang Normal University, Xinyang 464000, China; jiangyuling@xynu.edu.cn (Y.J.); guohao@xynu.edu.cn (H.G.); chenky@xynu.edu.cn (K.C.); 54838@xynu.edu.cn (X.F.); 0509@xynu.edu.cn (M.L.); 2 Henan Key Laboratory for Synergistic Prevention of Water and Soil Environmental Pollution, Xinyang Normal University, Xinyang 464000, China; 3 National Demonstration Center for Environmental and Planning, College of Geography and Environmental Science, Henan University, Kaifeng 475004, China; mjh@henu.edu.cn

**Keywords:** heavy metals, soil, rice, health risk assessment

## Abstract

The accumulation of potential toxic elements in agricultural soil and rice is of particular concern in China. However, studies on the risk assessment of these elements in regional soil–rice systems remain limited. The aim of this study is to evaluate the pollution status and potential health risk of potential toxic elements in typical paddy soil and rice in Henan Province. A total of 80 soil samples and corresponding rice samples were collected to determine the contents of Cd, Pb, As, Cr, Cu, Zn, and Ni, and to assess their potential health risks to local consumers. Results showed that the average contents of these elements in soils were below the national risk screening values in GB15618-2018. Only the average content of Cr in rice exceeded the limit in GB 2762-2022 specified by the national food safety standard. The rates of exceeding the limits for Cd, Pb, As, and Cr in rice samples were 13.89%, 15.28%, 15.28%, and 27.78%, respectively. The health risk assessment indicated that rice intake for both adults and children caused carcinogenic and non-carcinogenic health risks to varying degrees. Local residents are advised to purchase rice from outside the study area to meet their daily needs and strictly regulate the pollution of potential toxic elements within the area.

## 1. Introduction

Soil is a key element of life on Earth [1,2]. The rapid development of global urbanization, industrialization, and intensive agriculture, on the one hand, has promoted social and economic development and improved people’s quality of life; on the other hand, it has also led to the entry of many potential toxic elements polluting farmland soil, resulting in soil environmental deterioration and quality reduction [3]. Numerous studies indicated that large-scale farmlands, both domestically and internationally, are contaminated with potential toxic elements to varying degrees [4,5,6,7,8]. The 2014 National Soil Pollution Survey Bulletin reported that 19.4% of farmland soil points exceeded acceptable levels of potential toxic elements in China. Among these pollutants, Cd, Ni, and Cu were identified as the most prevalent contaminants [9]. News about food safety and human health problems caused by potential toxic element pollution in farmland soil is also endless, with “cadmium rice” and “cadmium wheat” being the most concerning [10,11,12]. Assessing the pollution levels of potential toxic elements in agricultural soil is essential, as it provides data to inform the selection of appropriate countermeasures.

In recent years, scholars have conducted extensive research on the soil–crop system, focusing on research hotspots such as potential toxic element content, pollution evaluation and source identification, and health risk evaluation, including crops such as rice, wheat, corn, vegetables, and melons [13,14,15,16]. The characteristics of pollution in soil and crops and the assessment of health risks are still the current research hotspots. However, previous studies have mostly focused on polluted sites or typical farmland blocks [17,18,19,20], and regional research work is insufficient. Conducting regional soil–crop potential toxic element collaborative health risk assessment research work is helpful for optimizing and adjusting the agricultural product structure to avoid foodborne hazards. Moreover, understanding the regional characteristics of pollution can support the development of more targeted environmental management policies tailored to the specific contamination profiles of different areas. The research area of Xinyang is located in the three mountain areas and one beach area of Henan Province, the Dabie Mountain concentrated poverty area belonging to the Qinling Mountains and Huai River dividing line, the dividing line between the north and the south, and the warm temperate zone and subtropical zone dividing line, with the geographical characteristics of the north and south compatibility [21]. Xinyang is the main rice producing area in Henan Province, and it is also a typical “rice-wheat” double cropping area. Although rice production is lower than corn production, it is also the second largest staple food of Henan’s people [22]. The rice from Xinyang is of great importance to the food supply not only of the people in Henan but also a majority of the Chinese population. Xinyang rice is regularly supplied to regions such as Guangxi and Guangdong. Rice is the most important food crop for the local people in Xinyang; many families eat rice three times a day. In addition, rice provides more than 70% of their daily calorie intake from food [23]. Consequently, the quality and safety of rice are closely related to human health and quality of life. However, the current pollution characteristics and health risks associated with the regional soil–crop system remain unclear. In view of this, this study aims to evaluate the potential toxic element pollution and health risk in typical paddy soil and rice in Henan Province in order to provide a scientific basis for the evaluation, governance, and risk management of potential toxic element pollution in regional soil and crops.

## 2. Materials and Methods

### 2.1. Overview of the Study Area

The study area is located in typical rice paddy region of Xinyang, Henan Province. The study area has a population of 6.4 million people [24]. Xinyang is situated at the junction of three provinces, Hubei, Henan, and Anhui, in the southern part of Henan Province. It is located between the northern foot of the Dabie Mountain range and the upper reaches of the Huai River. The geographical coordinates are between 113°45′ and 115°55′ east longitude and 31°23′ and 32°37′ north latitude. Xinyang has numerous rivers belonging to the Yangtz River and Huai River water systems. The soil types are mainly yellow-brown earths, lime concretion black soils, and paddy soil [25].

### 2.2. Sample Collection and Pre-Treatment

**Figure 1:**
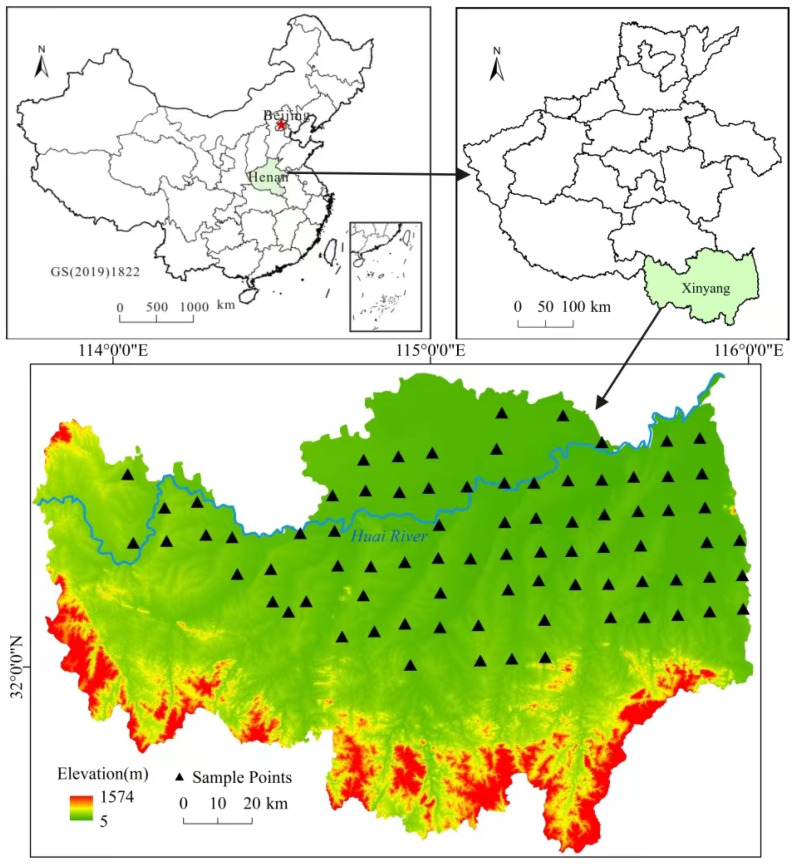
Sampling point distribution.

Uniform grid sampling was conducted using a 10 km × 10 km grid method, with soil samples collected near the center of each grid point. To avoid the strong impact of human activities on the soil, sampling points were located more than 5 km from cities, more than 2 km from townships, residential areas, transportation arteries, and industrial enterprises, more than 1 km from villages, and more than 200 m from farm roads and ditches. A total of 80 soil samples and corresponding rice samples were collected in the study area, and the sampling points are shown in Figure 1.

At the sampling points, researchers first selected representative 10 m × 10 m sampling units. Within each unit, in the area of 25 m^2^ around the sampling point, five surface soil subsamples were collected according to the “quincunx” method. After mixing the five subsamples, a representative soil sample of this location was formed. Plant debris, bricks, and pebbles were removed, and the remaining soil was reduced using the “quartering method” to obtain approximately 1 kg of soil for analysis. Rice samples were similarly collected through multi-point mixing.

Samples were naturally air-dried and passed through a 10-mesh nylon sieve with thorough mixing (for pH and cation exchange capacity (CEC) detection). Approximately 25 g of soil was randomly taken from around 30 points from the 10-mesh sample, ensuring that all particles passed through a 100-mesh nylon sieve (for potential toxic element and organic matter (OM) content detection) [26]. After drying, the rice samples were threshed by hand kneading the sample bags, and the plastic bags were sealed tightly. The pH in soil was measured using a PHSJ-3F pH meter with a soil-to-water ratio of 1:2.5 (*w*/*v*). The content of CEC in soil samples was measured by the EDTA-ammonium acetate exchange method [27]. The OM content was determined using the wet oxidation method with K_2_Cr_2_O_7_ and H_2_SO_4_ in an oil bath at 170–180 °C, followed by titration with calibrated FeSO_4_ [28]. To determine the contents of Cd, Pb, As, Cr, Cu, Zn, and Ni, HNO_3_-HF-HClO_4_ was used to digest soil samples [29], and HNO_3_-HClO_4_ was used to digest rice samples [30]. For the determination of As content in the samples, soil and rice samples were digested using aqua regia and HNO_3_-H_2_O_2_ systems, respectively, under a microwave digestion apparatus, and then determined with an atomic fluorescence spectrometer (AFS-3100, Beijing Haiguang, China). The detection and quantification limits were as follows: 0.0003 mg/kg and 0.0009 mg/kg for Cd, 0.004 mg/kg and 0.015 mg/kg for Pb, 0.0007 mg/kg and 0.003 mg/kg for As, 0.004 mg/kg and 0.014 mg/kg for Cr, 0.004 mg/kg and 0.015 mg/kg for Cu, 0.07 mg/kg and 0.25 mg/kg for Zn, and 0.004 mg/kg and 0.015 mg/kg for Ni.

During the measurement process, each batch of samples (soil samples and wheat samples) were quality-controlled, respectively, with standard sample spike recovery (GSS-2 and GSB-24), parallel samples, and blank samples. Each batch includes three standard samples and three blank samples, while parallel samples constitute 20% of the batch. The recovery rates (84.3–108.2%) and the relative errors of parallel samples (0.5%) are all within the acceptable range.

### 2.3. Bioaccumulation Factor

The bioaccumulation factor (BAF) reflects the plant’s ability to absorb pollutants from the surrounding soil [31]. The higher the BAF value, the stronger the ability of rice to accumulate potential toxic elements; a lower BAF indicates that rice has a weaker ability to accumulate potential toxic elements and stronger resistance to pollution [32]. BAF was calculated as follows:

(1) $$BAF=\frac{C_{rice}}{C_{soil}}$$

where *C_rice_* represents the element content in rice (mg/kg) and *C_soil_* is the element content in soil (mg/kg).

### 2.4. Health Risk Assessment Methods

The human body is exposed to different degrees of carcinogenic and non-carcinogenic risks of potential toxic elements through ingesting rice. Cd, Pb, As, Cr, Cu, Zn, and Ni all pose a non-carcinogenic health risk, among which As, Cd, Ni, and Cr also pose a carcinogenic risk. The daily exposure amounts, carcinogenic and non-carcinogenic risk characterization models, and parameter selections for various potential toxic elements are shown in Table 1 and Table 2 [33,34,35,36,37,38].

In the formulas in Table 1, *ADD_i_* is the average daily intake of element *i* through rice ingestion, mg·(kg·d)^−1^; *C_i_* is the content of a potential toxic element in rice, mg·kg^−1^; *HQ_i_* is the non-carcinogenic health risk index of potential toxic element *i*; *HI* is the total non-carcinogenic risk index; *CR_i_* represents the single health risk index of a carcinogenic potential toxic element *i*; *TCR* is the total carcinogenic risk index caused by a carcinogenic potential toxic element. When *HQ_i_* or *HI* is less than 1, the non-carcinogenic health risk can be ignored; when it is greater than 1, this indicates the presence of non-carcinogenic health risks. When *CR_i_* or *TCR* is lower than 1 × 10^−6^, the carcinogenic risk is negligible; when 1 × 10^−6^ < *CR_i_* or *TCR* < 1 × 10^−4^, the carcinogenic risk is in an acceptable range; when *CR_i_* or *TCR* is higher than 1 × 10^−4^, consuming rice has a strong carcinogenic risk and needs to be strictly controlled [40].

Referring to the US EPA [33,34] and the site environmental assessment guidelines [41], as well as related research results [42,43,44], the values of *RfD* and *SF* are shown in Table 3.

## 3. Results and Discussion

### 3.1. Contents of Potential Toxic Elements in Paddy Soil

The Table 4 presents the contents of potential toxic elements and selected soil properties. The contents of Cd, Pb, As, Cr, Cu, Zn, and Ni in soil samples ranged from 0.08 to 0.31, 18.37 to 36.70, 0.01 to 26.69, 51.52 to 147.43, 17.39 to 47.09, 7.11 to 182.25, and 17.36 to 60.01, with average contents of 0.14, 23.98, 6.19, 71.36, 26.57, 57.95, and 30.39 mg/kg, respectively. Compared with the background values of soil (A layer) in Henan Province, the average contents of Cd, Pb, Cr, Cu, and Ni were higher than the background values, equivalent to 2.00, 1.8, 1.13, 1.33, and 1.11 times the background values, respectively. The average contents of As and Zn were both lower than their background values, equivalent to 63.16% and 92.72% of their background values, respectively. It is observable that there is a varying degree of accumulation of elements such as Cd and Cu in the soil of the rice-growing areas of Xinyang. Compared with the risk screening values in GB15618-2018 [45], the average contents of the seven potential toxic elements in the soil were below the screening values; the maximum contents of each potential toxic element also did not exceed the screening values, so each element had an excess rate of 0. This indicates that the environmental quality of the soil in the study area is relatively good.

The coefficient of variation (CV) of potential toxic element content reflects the degree of spatial variation between sampling points in the study area. A CV of less than 20% indicates a relatively weak degree of variation, 20% ≤ CV ≤ 50% indicates a medium degree of variation, and 50% < CV ≤ 100% indicates a strong degree of variation [46]. The CVs for As and Zn were relatively large, at 56.1% and 60.47%, respectively, both falling into the category of strong variation; the CVs for Cd, Pb, Cr, Cu, and Ni were 19.47%, 12.93%, 21.93%, 17.54%, and 19.63%, respectively, with Cr being the only one in the moderate variation category, and all others showing weak variation. The relatively large variation ranges in the contents of As and Zn may be influenced by human activities [47].

The soil pH ranged from 4.77 to 7.14, with an average pH of 6.34 (Table 4), indicating slightly acidic conditions. The SOM content varied between 6.55 and 38.55 g/kg, with an average content of 26.25 g/kg, suggesting a medium level of SOM content (20–30 g/kg) in the region [48]. The CEC content ranged from 8.93 to 47.50, with an average content of 22.88 cmol(+)/kg, classifying the soil as having medium fertility. The CVs for soil pH, SOM, and CEC were 8.17%, 34.71%, and 36.30%, respectively. Different fertilization methods across farmlands may result in rice cultivation having a relatively minor effect on soil pH, while significantly impacting soil SOM and CEC.

### 3.2. Contents of Potential Toxic Elements in Rice

According to the statistics for the potential toxic element contents in rice (Table 5), the average contents of Cd, Pb, As, Cr, Cu, Zn, and Ni in rice were 0.11, 0.14, 0.34, 1.46, 3.61, 18.05, and 8.79 mg/kg, respectively, in descending order as Zn > Ni > Cu > Cr > As > Pb > Cd, with the nutritional element Zn having the highest content and the more toxic elements Pb and Cd having the lowest content. Pb and Cr have a larger fluctuation range, at 0.01~2.49 mg/kg and 0.01~11.68 mg/kg, respectively, with corresponding CVs reaching 224.17% and 177.52% (Table 5), indicating a large degree of spatial variation. Pb and Cr are considered to have very strong variation, Cd, Cu, As, and Ni show strong variation, and Zn shows moderate variation. The average content of Cr in rice was significantly higher than the permissible limits in China of GB 2762-2022 [49], exceeding the standard by 1.46 times, while the average contents of the other elements were lower than the standard values. The rates of exceeding the limits for Cd, Pb, As, and Cr in rice samples were 13.89%, 15.28%, 15.28%, and 27.78%, respectively. The results showed that the Cd, Pb, As, and Cr in rice were seriously polluted, which should be paid attention to.

### 3.3. Correlation Analysis of Potential Toxic Elements in Soil and Rice

Based on the analysis of potential toxic elements in soil and rice, Cd and Cu accumulated in soil to varying degrees, and the Cd, Pb, As, and Cr in rice were seriously polluted. The Pearson correlation analysis results show (Table 6) that there were no significant correlations between the contents of potential toxic elements in the soil and corresponding potential toxic elements in the rice, and all correlation coefficients were low. The correlation coefficients for Cd, Cu, Zn, and Ni were negative. Studies have shown that low contents of potential toxic element stress have a certain role in promoting root vitality; but, with the continuous increase in potential toxic element contents, the ability of crops to absorb and transport potential toxic elements will gradually decrease, preventing the further accumulation of potential toxic elements in plants. In addition, it can be seen that the contents of potential toxic elements in the soil of the study area did not exceed the screening values, whereas the contents of potential toxic elements in rice exceeded the standard values seriously. The absorption of potential toxic elements by crops is influenced by multiple factors, making it insufficient to rely solely on the soil’s pollution status to assess the accumulation of these elements in crops.

### 3.4. Bioaccumulation of Potential Toxic Elements in Rice

The bioaccumulation factors (BCF_S_) for Cd, Pb, As, Cr, Cu, Zn, and Ni were 2.27~277.74%, 0.02~9.81%, 0.02~51.75%, 0.06~17.16%, 3.76~95.32%, 8.37~512.99%, and 0.32~25.66%, respectively (Table 7); the BCF_S_ of Cd, Cu, and Zn varied widely. The average BCF_S_ for Cd, Pb, As, Cr, Cu, Zn, and Ni were 86.08%, 0.57%, 7.26%, 1.91%, 14.11%, 51.03%, and 5.72% (Table 3). The accumulation capacity of these potential toxic elements in rice followed this order: Cd > Zn > Cu > As > Ni > Cr > Pb [48]. The BCF_S_ of potential toxic elements in rice were related to plant growth characteristics and the chemical form of potential toxic elements, among other factors. Zn and Cu are trace elements required for plant growth, playing an important role in physiological activities such as chlorophyll formation, respiration, and photosynthesis in plants, and thus are relatively more accumulated in plant bodies [50]. Cd mainly exists in the form of an exchangeable state in acidic paddy fields, with a strong migration ability, and rice has a higher accumulation capacity for Cd. Cr, Ni, Pb, and As, which are mostly present in the soil in the form of residue, especially the stable form of Cr, which accounts for more than 85%, making it difficult for plants to absorb [51]. In addition, the physicochemical properties of the soil and rice varieties also affect the absorption of potential toxic elements by rice to a certain extent.

### 3.5. Human Health Risk Assessment 

According to the dietary guidelines for Chinese residents and the survey of the dietary status of local residents, rice is the primary food crop in the study area. In conjunction with the latest Henan statistical yearbook of 2023 and the actual situation of the local area, the average daily rice intake is 437.56 g for adults and 145.86 g for children [24].

**Figure 2:**
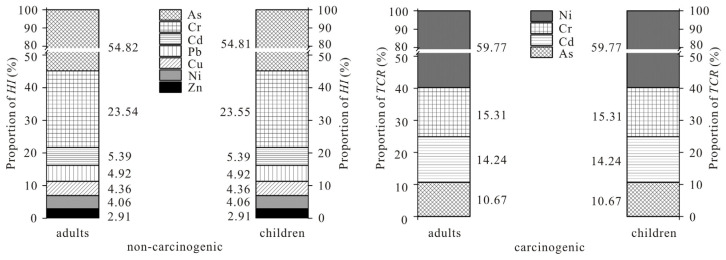
Contribution rates of different potential toxic elements.

The non-carcinogenic risks (Table 8) associated with exposure to potential toxic elements through rice ingestion revealed that the HQ of children was higher than that of adults. Except for HQ_As_, HQ_Cr_, which was significantly greater than 1, the HQ of other potential toxic elements in adults was lower than 1. The HQ values of Cd, As, and Cr were higher than 1 in children. Based on the HQ values for different elements, the HQ value of As was the largest among adults and children (7.58 and 10.58, respectively). Judging from the results of element contribution rates (Figure 2), the HQ value of As constituted 54.82% of the total HI of adults and 54.81% for children, indicating that As is the main non-carcinogenic potential toxic element. Followed by Cr, the HQ_Cr_ of adults and children was 3.25 and 4.54, respectively, comprising over 23.5% of the total HI. This indicated that As, Cr, and Cd are the primary non-carcinogenic risk factors in the study area. Enhancing the pollution control of As, Cr, and Cd is essential to safeguard residents’ health. The total HI for adults and children exposed to potentially toxic elements through rice consumption was 13.82 and 19.30, respectively (Table 8), both much higher than 1, indicating that the non-carcinogenic health risk is relatively serious and requires attention.

The carcinogenic risk assessment results indicated that both the CR and TCR are higher for children than adults (Table 9). The TCR for adults and children from rice intake was 3.20 × 10^−2^ and 4.50 × 10^−2^, respectively, which was far greater than the limit set by the US EPA (1 × 10^−4^), indicating a very serious carcinogenic risk. CR_Cd_, CR_As_, CR_Cr_, and CR_Ni_ were higher than 1 × 10^−4^, with CR_Ni_ contributing the largest, accounting for 59.77% of the total TCR for both adults and children (Figure 2), and was the most significant carcinogenic potential toxic element. Secondly, CR_Cr_ accounted for 15.31% of the total TCR of adults and children.

The results indicated that rice consumption poses varying degrees of carcinogenic and non-carcinogenic health risks to both adults and children. Similar health risks associated with rice consumption have been reported in other domestic provinces, such as Hunan [52] and Guizhou [53], as well as in countries like Thailand [54] and Iran [55]. The contribution rates of potential toxic elements vary significantly across regions. According to local conditions, the targeted control of heavy metal pollution in each region should be implemented. It is also recommended that local residents reduce their rice intake and consider purchasing food from outside the study area.

### 3.6. Uncertainty Analysis

The health risk assessment results of human exposure to potential toxic elements were influenced by many factors, including the accuracy of exposure parameters, the stability of the model, the representativeness and uniformity of the selected samples, and the overall sampling results [38]. Consequently, risk assessment results often exhibit significant uncertainty. The small sample size of this study may introduce a degree of randomness to the results. In addition, there were differences between adults and children in terms of dietary habits, living environments, and physiological characteristics. The health risk assessment results are mainly calculated based on the data provided by the statistical yearbook of Henan Province, the US EPA, and China’s population exposure parameter manual. Thus, the results may not fully reflect the specific conditions of the study area, potentially leading to deviations in the assessment. The health risk assessment model also has a certain degree of uncertainty. Despite these limitations, this study provides a valuable assessment of the risks posed by exposure to potential toxic elements through rice consumption. These findings offer an important foundation for prioritizing health and environmental risk management efforts in the study area.

**Figure 3:**
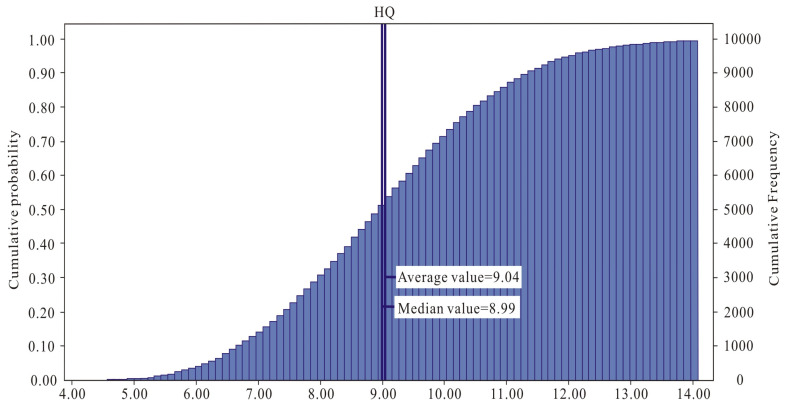
Distribution of non-carcinogenic risk for children exposed to As through rice ingestion.

Monte Carlo simulation was employed to reduce the uncertainty of the risk assessment results. To assess the non-carcinogenic risk of As exposure in children through rice consumption, a comprehensive analysis was conducted. This analysis involved repeatedly calculating the risk 10,000 times, taking into account various factors such as the distribution characteristics and parameter settings of children’s rice intake, body weight, and the As content in rice. By integrating these factors, this study aimed to provide a detailed and accurate estimation of the potential health risks associated with arsenic exposure in children. A sensitivity analysis using Crystal Ball software (version 11.1.30) revealed that the As concentration in rice is the most significant factor affecting the non-carcinogenic risk of human exposure, with a sensitivity of 96%. The mean and median values of the HQ_As_ were 9.04 and 8.99 (Figure 3), respectively, which are relatively close to the value of 10.58 obtained using the health risk evaluation model recommended by the US EPA. This indicated minimal bias in the non-carcinogenic risk assessment of children’s As exposure from rice consumption in this study. It showed that local residents face a significantly higher health risk, which requires more attention.

## 4. Conclusions

The average contents of Cd, Pb, As, Cr, Cu, Zn, and Ni in soil were below the screening values in GB 15618-2018, indicating that the environmental quality of the soil in the study area is relatively good. But, compared with the limits in GB 2762-2022 specified by the national food safety standard, the rates of exceeding the limits for Cd, Pb, As, and Cr in rice samples were 13.89%, 15.28%, 15.28%, and 27.78%, respectively, indicating that Cd, Pb, As, and Cr in rice were seriously polluted. The accumulation capacity of these potential toxic elements in rice decreased in the following order: Cd > Zn > Cu > As > Ni > Cr > Pb. The health risk assessment indicated that rice intake for both adults and children caused carcinogenic and non-carcinogenic health risks to varying degrees, with the risk for children being higher than that for adults. It is strongly recommended that local residents reduce their consumption of locally sourced rice. Instead, they should consider purchasing rice from outside the region to meet their dietary needs. Additionally, it is crucial to implement stringent measures to control and monitor the pollution of potential toxic elements in the area.

## Figures and Tables

**Table 1:** Assessment models of human health risk.

Health Risks		Compartment	Equations
Exposure pathway	Ingestion	Rice	${ADD}_{i}=\frac{C_{i}\cdot I_{i}\cdot EF\cdot ED}{BW\cdot AT}$
Non-carcinogenic health risk	Hazard quotient	Cd, Pb, As, Cr, Cu, Zn, Ni	$HQ_{i}=\frac{ADD_{i}}{RfD_{i}}$
	Health risk		$HI=\sum_{1}^{7}{HQ}_{i}$
Carcinogenic health risk	Carcinogenic risk of single metals	Cd, As, Cr, Ni	$CR_{i}=ADD_{i}\times SF_{i}$
	Total carcinogenic risk		$TCR=\sum_{i=1}^{4}{CR}_{i}$

**Table 2:** Exposure parameters for potential toxic elements in rice.

Parameter Symbols	Parameters	Values (Adults)	Values (Children)
*BW* [38]	Body weight/kg	62.8	15
*I_i_* [24]	Rice ingestion rate/g·d^−1^	0.1	0.2
*ED* [31]	Lifetime exposure duration/a	30 (non-carcinogenic), 70 (carcinogenic)	6 (non-carcinogenic), 70 (carcinogenic)
*EF* [31]	Exposure frequency/d·a^−1^	365	365
*AT* [39]	Exposure time/d	*AT* = *ED* × 365 (non-carcinogenic) *AT* = 25,550 (carcinogenic)	*AT* = *ED* × 365 (non-carcinogenic) *AT* = 25,550 (carcinogenic)

**Table 3:** Values of *RfD* and *SF* for ingestion of rice.

	Cd	Pb	As	Cr	Cu	Zn	Ni
*RfD* [33]	1.00 × 10^−3^	1.40 × 10^−3^	3.00 × 10^−4^	3.00 × 10^−3^	0.04	0.30	0.02
*SF*	6.1 [43,44]	NA	1.50 [33]	0.50 [34]	NA	NA	1.70 [34]

NA—not applicable.

**Table 4:** Contents of potential toxic elements in soil (mg/kg).

	pH	SOM	CEC	Cd	Pb	As	Cr	Cu	Zn	Ni
Min	4.77	6.55	8.93	0.08	18.37	0.01	51.52	17.39	7.11	17.36
Max	7.14	38.55	47.50	0.31	36.70	26.69	147.43	47.09	182.25	60.01
Mean	6.34	26.25	22.88	0.14	23.98	6.19	71.36	26.57	57.95	30.39
SD	0.52	0.91	8.31	0.03	3.10	3.47	15.65	4.66	35.04	5.97
CV (%)	8.17	34.71	36.30	19.47	12.93	56.01	21.93	17.54	60.47	19.63
Background value [46]	_	_	_	0.07	22.3	9.8	63.2	20.0	62.5	27.4
Risk screening value [45]	_	_	_	0.4	100	30	250	50	200	70
Exceeded screening (%)				0	0	0	0	0	0	0

**Table 5:** Contents of potential toxic elements in rice (mg/kg).

	Cd	Pb	As	Cr	Cu	Zn	Ni
Min	0.003	0.01	0.002	0.01	1.15	7.44	0.11
Max	0.33	2.49	1.76	11.68	27.88	40.41	8.79
Mean	0.11	0.14	0.34	1.46	3.61	18.05	1.68
GB 2762-2022	0.2	0.2	0.5	1.0	——	——	——
SD	0.08	0.32	0.22	2.59	3.25	6.65	1.27
CV (%)	70.70	224.17	65.66	177.52	90.00	36.84	75.28
Exceeded–standard (%)	13.89	15.28	15.28	27.78	——	——	——

**Table 6:** Results of Pearson correlation analysis of potential toxic elements in soil and rice.

	Cd-r	Pb-r	As-r	Cr-r	Cu-r	Zn-r	Ni-r
Cd-s	−0.127	0.014	−0.051	−0.150	−0.084	0.053	−0.068
Pb-s	−0.133	0.064	−0.059	−0.046	0.047	−0.052	0.063
As-s	−0.057	0.119	0.012	−0.024	−0.149	−0.070	−0.196
Cr-s	−0.066	0.067	−0.066	0.148	0.035	−0.096	−0.021
Cu-s	−0.208	−0.047	−0.121	−0.026	−0.011	−0.173	−0.033
Zn-s	0.013	−0.058	−0.116	−0.165	−0.017	−0.016	−0.055
Ni-s	−0.174	0.114	0.032	0.093	0.017	−0.113	−0.042

**Table 7:** Statistical results of soil–rice bioaccumulation factors (%).

	Cd	Pb	As	Cr	Cu	Zn	Ni
Min	2.27	0.02	0.02	0.06	3.76	8.37	0.32
Max	277.74	9.81	51.75	17.16	95.32	512.99	25.66
Mean	86.08	0.57	7.26	1.91	14.11	51.03	5.72

**Table 8:** Non-carcinogenic health risk assessment for adults and children.

	*HQ*							*HI*
	*HQ*_Cd_	*HQ*_pb_	*HQ*_As_	*HQ*_Cr_	*HQ*_Cu_	*HQ*_Zn_	*HQ*_Ni_	
Adults	0.74	0.68	7.58	3.25	0.60	0.40	0.56	13.83
Children	1.04	0.95	10.58	4.54	0.84	0.56	0.78	19.30

**Table 9:** Carcinogenic risk index for adults and children.

	*CR_Cd_*	*CR_As_*	*CR_Cr_*	*CR_Ni_*	*TCR*
Adults	4.50 × 10^−3^	3.40 × 10^−3^	4.90 × 10^−3^	1.90 × 10^−2^	3.20 × 10^−2^
Children	6.30 × 10^−3^	4.70 × 10^−3^	6.80 × 10^−3^	2.70 × 10^−2^	4.50 × 10^−2^

## Data Availability

Data are contained within the article.
